# Prevalence and factors associated with early childbearing in sub-saharan Africa: evidence from demographic and health surveys of 31 countries

**DOI:** 10.1186/s12905-023-02581-z

**Published:** 2023-08-14

**Authors:** Liness Shasha, Million Phiri, Sibongile Namayawa, Milika Sikaluzwe, Chola Nakazwe, Musonda Lemba, Mikidadi Muhanga

**Affiliations:** 1 Department of Population Studies, School of Humanities and Social Sciences, University of Zambia, Lusaka, Zambia; 2 Demography and Population Studies Programme, Schools of Public Health and Social Sciences, University of the Witwatersrand, Johannesburg, South Africa; 3 Zambia Statistics Agency, Lusaka, Zambia; 4 School of Public Health, University of Zambia, Lusaka, Zambia; 5 Department of Development and Strategic Studies, College of Social Sciences and Humanities - Sokoine, University of Agriculture, Morogoro, Tanzania

**Keywords:** Women, Adolescent health, Early childbearing, Reproductive health, Sub-Saharan Africa

## Abstract

**Background:**

Early childbearing remains a public health concern in sub-Saharan Africa (SSA) because it has substantial implications for women’s and children’s health and population control. However, little is known about recent changes in early childbearing in the region following the implementation of the Family Planning 2020 initiative (FP2020) national-level interventions. Thus, this study examined factors associated with early childbearing among women in SSA.

**Methods:**

The study used data from the most recent Demographic and Health Surveys conducted in 31 countries in sub-Saharan Africa between 2010 and 2021. The analysis included a pooled sample of 54,671 parous young women aged 20–24 years. A multivariable binary logistic regression model was used to examine the association between early childbearing and individual and household-level factors. All analyses were weighted to account for complex survey design.

**Results:**

The study shows that the mean prevalence of early childbearing was high in SSA at 39% (95% CI: 35, 43). Chad had the highest prevalence of early childbearing, 62% (95% CI: 60, 64) while Rwanda had the lowest prevalence of 13% (95% CI: 11, 15). Completing secondary school (aOR = 0.57; 95% CI: 0.52, 0.62) or attaining tertiary level education (aOR = 0.32; 95% CI: 0.22, 0.45), first sexual debut in the age range 15–24 years (aOR = 0.15; 95% CI: 0.14, 0.16) and desire for a small family size (aOR = 0.63; 95% CI: 0.58, 0.69) were associated with reduced odds of early childbearing among young women in SSA.

**Conclusion:**

The study has established that the prevalence of early childbearing is high in SSA. Level of education, age at first sexual debut, household size, and desired family size are associated with early childbearing in SSA. Governments of SSA countries should enhance sexual and reproductive health interventions to change reproductive behaviour, particularly in adolescents and young women.

## Introduction

Early childbearing refers to a practice of a woman giving her first birth at a relatively young age, that is an age before 18 years [1, 2]. Early childbearing can pose health risks to both the mother and child, as well as social and economic challenges for the family. The prevalence of early childbearing is considered being high in many sub-Saharan (SSA) countries [3–8]. This is one reason why the SSA region has persistently high fertility and population growth. The region, according to the United Nations estimates, is reported to have the highest total fertility rate (TFR) in the world, at 4.7 births per woman during the period 2015 to 2020 [9, 10]. As a result, it is anticipated that the continent’s population would grow from 1 billion in 2015 to over 2 billion in 2050 and close to 4 billion in 2100 [10]. It is expected that the region’s population will experience a significant increase in the negative impacts on human welfare and the environment [11, 12]. Fertility is one of the three main factors in population dynamics that affect SSA population size and composition [13–15]. One of the most common findings in demographic studies from SSA has been differences in fertility levels and behaviour across sub-regions, population strata, and characteristics [13, 16].

Although the reproductive health outcomes in SSA have significantly improved, the total fertility rate is still high compared to other developing regions [17–20]. These improvements include a decrease in maternal and infant mortality; a rise in the prevalence of contraception; and increased use of health services by married women [17, 19, 21, 22]. The high proportion of women who start childbearing in adolescence age is one of the main reasons for the high fertility rates in most countries in the region [20, 23]. Other factors contributing to the high fertility rate in SSA are early and universal marriage, as well as the desire for males for both cultural (performing rites) and economic (to reap immediate financial benefits and ensure old age security) reasons [4, 23, 24]. To better understand the causes of early childbearing in SSA and tailor reproductive health programmes to these needs, more research and inquiry are required.

Most governments in SSA have made significant investments in family planning programming, provision of education, and strengthening frameworks to discourage early sexual debut and child marriage. However, reducing the prevalence of early childbearing in SSA will require strengthening of sexual and reproductive health interventions to ensure adequate access and utilisation of family planning services, especially among adolescents and vulnerable young women. Recent demographic data are expected to reveal new patterns of early childbearing levels in the region. Nevertheless, it should be emphasised that some countries in SSA have not carried out recent Demographic and Health Surveys. As a result, this study is limited in its ability to thoroughly investigate emerging trends in early childbearing rates across the region.

Considering that SSA has the highest fertility rate in the world, early childbearing has the potential to disrupt a girl’s education and limit her future social and economic opportunities. Studying the factors associated with early childbearing can inform designing of interventions to promote comprehensive sexuality education, reproductive health services, and programmes that empower girls to delay childbearing and pursue education and career goals. Thus, this study used recent fertility data from demographic and health surveys conducted in 31 SSA countries between 2010 and 2021 to establish differentials and examine factors associated with early childbearing in SSA. It should be noted that a comprehensive understanding of the factors associated with early childbearing is essential for designing appropriate interventions to further reduce fertility in SSA. The findings could also inform the strengthening of existing sexual reproductive health policies and strategies aimed at increasing access and utilisation of sexual reproductive health care services in SSA.

### Theoretical framework

Theoretically, early childbearing can be understood within the theoretical underpinnings of the Classical Demographic Transition theory and the Empowerment theory. The two theories contribute valuable insights to understanding the complex factors that explain early childbearing experience among women in SSA. The Classical Demographic Transition Theory was initially proposed by Warren Thompson in 1929 and later refined by Frank W. Notestein in the mid-20th century [25]. This theory suggests that countries go through a predictable sequence of demographic changes as they undergo economic and social development [25–27]. One of the central assumption is that birth rates decline due to various factors associated with modernisation and socio-economic development [26, 27]. These factors often include increased urbanisation, improved education for women, greater access to contraception, and changes in cultural and social norms [28, 29]. The Demographic Transition Model remains a valuable framework for understanding historical population trends and provides insights into the potential demographic changes that countries may experience as they undergo social and economic transformations.

The Empowerment theory is a sociological and psychological framework that focuses on enhancing the power and agency of individuals and groups in order to promote social change and improve their well-being. It emerged as a response to traditional deficit-oriented approaches that viewed individuals and communities as passive recipients of services or interventions [30, 31].

Both theories hypothesise that social development such as improvements in female education and women’s empowerment are key in influencing early childbearing among women of reproductive age [9, 17, 32, 33]. Women who have low levels of education and low economic opportunities may be more likely to experience early childbearing [34, 35]. Further, structural inequalities such as living in rural areas may present women with less access to family planning services, which increases their risk to early pregnancy. Gender norms may also limit women’s agency and decision-making power to make informed choice about their reproductive goals, hence contributing to early childbearing [36]. Economic vulnerability and limited opportunities for education and employment may push women towards early childbearing as they may perceive motherhood as a more viable path than pursuing other life goals [37, 38].

Based on the theoretical framework used in this study, it is expected that women who belong to vulnerable groups such as those with low level of education, reside in rural regions, the poorest, and know the least about contraceptives are more likely to fall pregnant during adolescence stage [32, 33, 39, 40]. Therefore, identification of the risk factors linked to experience of early childbearing and understanding of the framework through which these factors operate, as well identifying which groups of women are at risk of experiencing early childbearing in SSA, is key. This information is crucial for designing reproductive interventions aimed at controlling high fertility in the region.

## Methods

### Data source

This study used secondary data from the Demographic and Health Survey (DHS) conducted in 31 SSA countries between 2010 and 2021.The Demographic and Health Survey (DHS) is a nationwide survey that is carried out across low-and middle-income countries every four or five years [41] and collects data on some fertility-related indicators such as marriage and sexual activity, fertility, and family planning and maternal health. Specifically, the study used the women’s files (IR) which contain responses of women aged 15–49. A stratified two-stage sampling approach was employed in selecting the sample for each survey. A pooled sample of 54,671 young women aged 20–24 years, who had ever given birth during their lifetime and had complete cases on all the variables of interest were included in the analysis. We excluded from the analysis all women aged 15–19 because they still had a risk of either experiencing or not experiencing early childbearing. The data analysed in this study is available in the public domain at (https://dhsprogram.com/). The sample description information is provided in Table 1.

**Table 1:** Description of study countries, samples and region (n = 54,671)

Country/DHS year	DHS year	Young women 20–24 interviewed	Sample (N)	Region
Angola	2015-16	3,060	2,476	Southern Africa
Benin	2015	2,916	1,942	West Africa
Burkina Faso	2010	3,243	2,461	West Africa
Burundi	2010	3,250	1,691	East Africa
Cameroon	2018	2,463	1,577	Central Africa
Chad	2014-15	2,995	2,447	Central Africa
Comoros	2012	987	393	Southern Africa
Congo Democratic Republic	2013-14	3,680	2,646	Central Africa
Cote d’Ivoire	2011-12	1,987	1,384	West Africa
Ethiopia	2016	2,903	1,574	East Africa
Gambia	2019	2,082	1,099	West Africa
Ghana	2014	1,571	795	West Africa
Guinea	2018	1,706	1,151	West Africa
Kenya	2014	5,405	3,661	East Africa
Lesotho	2014	1,300	834	Southern Africa
Liberia	2019-20	1,408	1,110	West Africa
Malawi	2015-16	5,094	3,999	Southern Africa
Mali	2018	1,907	1,449	West Africa
Mauritania	2019-21	2,731	1,361	West Africa
Mozambique	2011	2,468	2,004	Southern Africa
Namibia	2013	1,720	1,056	Southern Africa
Nigeria	2018	6,844	4,320	West Africa
Rwanda	2019-20	2,424	984	East Africa
Senegal	2019	1,623	930	West Africa
Sierra Leone	2019	2,602	1,765	West Africa
South Africa	2016	1,408	819	Southern Africa
Tanzania	2015-16	2,467	1,720	East Africa
Togo	2013-14	1,604	928	West Africa
Uganda	2016	3,782	2,831	East Africa
Zambia	2018	2,687	2,028	Southern Africa
Zimbabwe	2015	1,782	1,236	Southern Africa

### Study measurements

#### Outcome variable

The outcome variable for this study was early childbearing. The outcome was measured using the DHS variable age at first birth (v212). Age at first birth in the DHS is defined as the “age at which a woman first had a live birth” [42]. The DHS collected the variable “age at first birth” in retrospect, that means the women were asked to report an event which already happened. Thus, the current age of a young woman at the time of the interview and when she first gave birth are different. During the DHS surveys, the data on age at first birth was collected from all eligible women who reported ever having a live birth. These women were asked to state the age at first birth in completed years. The original variable in the DHS dataset was classified as a continuous response category. To facilitate binary logistic regression analysis, a binary outcome was then created from the initial variable with the code “1” representing age at first birth below the age of 18 years, which was classified as early childbearing. Then women who had their first birth in the age group of 18–24 years were coded as “0” representing none early childbearing. The age cut-off was derived based on the United Nations definition of a child as a person who is below the age of 18 years [43, 44].

#### Independent variables

The main independent variables for this study included age; place of residence; education level; employment status; household size and household wealth. Other predictors included age at first sex, exposure to media and exposure to FP messages. The variables were selected based on their importance in explaining experience of childbearing, as reported by prior studies conducted in SSA counties [7, 45–48]. Variables such as education, employment and wealth status have been reported to reduce the risk of early childbearing among adolescents. This is because they have the potential to provide women with knowledge about contraceptive method use, autonomy, financial stability, expanded aspirations, and the ability to challenge social norms [46–51]. These factors have the potential to reduce the risk of early childbearing by empowering adolescents to make informed sexual reproductive health decisions to delay sexual debut or use contraception to prevent pregnancy.

We classified these variables as individual and household-level determinants. Individual-level factors included the age of young women categorised as [20–24]; age at first marriage (below 15 years, 15–19 years, and 20–24 years); and age at first sex (below 15 years, 15–24 years). Other individual variables included education level (no education, primary, secondary, and tertiary); working status (unemployed, employed); knowledge of any FP method (knows no method, knows modern method); exposure to media family planning message (yes, no); visited by a community health worker (yes, no). The following household-level factors were included; place of residence (rural/urban); wealth index (poor, moderate, rich), and household size (1–3, 4–6, 7+).

### Statistical analysis

Stata software version 17.0 was used for all statistical analyses. Chi-square test was used to analyse associations between the dependent and independent variables. A multivariable binary logistic regression model was used to investigate the association between early childbearing and each individual and household-level factors. The “svy” command in Stata software was used to perform weighted analysis to account for the Demographic and Health Survey’s sampling weights and complex survey design. Sample weight equalisation was performed to give equal weight to each survey, such that if one survey had a large sample, it did not predominate the pooled results. Further, this process ensured that the sample weights were aligned to the clusters and strata within each country. This process addressed the variations in sampling fractions used by DHS in different countries. To examine the determinants of early childbearing among young women, a multivariate binary logistic regression model was conducted on a pooled dataset. The adjusted odds ratios (aOR) with 95% confidence intervals (CIs) were used to report results. To assess multicollinearity among independent factors, the variance inflation factor (VIF) was used. There were no concerns with multicollinearity in any of the variables (all VIF < 5).

### Ethical approval

Permission to use the data was obtained from the DHS program. All data used did not contain any identifying information. The country’s respective DHS Biomarker and survey protocols were approved by each country’s Ethical Review Body, the Institutional review board of ICF/Macro International, and where applicable the Research Ethics Review Board of the Centers for Disease Control and Prevention (CDC) Atlanta. Thus, all data collection methods were carried out under relevant ethical guidelines and regulations. The DHS protocols ensured that all participants older than 18 years who were enrolled in the DHS gave their informed consent during enumeration. Additionally, parents or guardians of all participants aged 15 to 17 gave informed consent before the legal minors were asked for their assent.

## Results

### Prevalence of early childbearing among young women in SSA countries

**Fig. 1:**
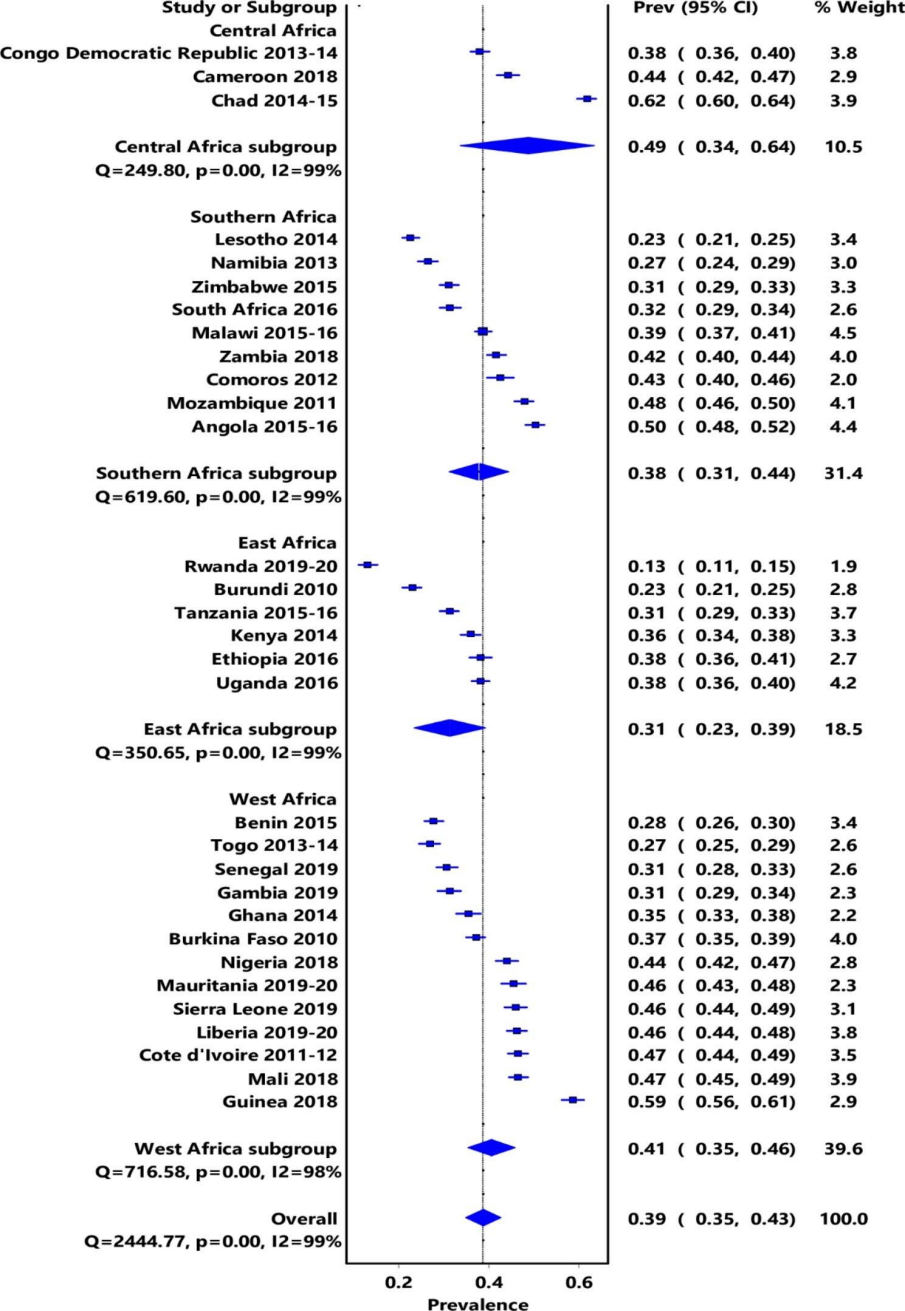
Prevalence of early childbearing among young women in SSA countries

Figure 1 shows the prevalence of early childbearing among young women in 31 SSA countries. The overall prevalence of early childbearing among young women in 31 SSA countries considered in this study was 39% (95% CI: 35, 43). In terms of country-based analysis, Chad 62% (95% CI: 60, 64) had the highest prevalence of early childbearing, while Rwanda 13% (95% CI: 11, 15) had the lowest prevalence. Sub-regional analysis shows that adolescent childbearing prevalence is highest in Central Africa, at 49% (95% CI: 34, 64) followed by West Africa, 41% (95% CI: 35, 46) and lowest in East Africa, 31% (95% CI: 23, 39).

### Distribution of early childbearing by background variables in SSA countries

Table 2 shows the distribution of early childbearing among young women by background variables. The highest prevalence of early childbearing was reported among young women aged 20 years (47.9%), those living in rural areas (41.1%), young women with no education (49.6%), those from poor households (43.7%) and young women who did not know any modern contraceptive methods (56.1%). The highest prevalence of early childbearing was also found among young women who had their first sexual debut early or were married before the age of 15 (75.5% and 89.6%), respectively. Furthermore, young women who belonged to households with 4–5 family members (46.3%), desired 6 or more children (49.5%), and those who had no exposure to family planning messages (41.3%) had the highest prevalence of early childbearing. The chi-square test results showed statistically significant associations between all the background variables and early childbearing except for community health worker visit, which showed no significant association with early childbearing.

**Table 2:** Percent distribution of young women (20–24) years who gave birth before age 18 years by background characteristics, SSA DHS Data (N = 54,671)

Early childbearing			
Background Characteristics	Number	Percent	P-value
**Age**			0.000***
20	5228	47.9	
21	3448	39.7	
22	4603	40.6	
23	3945	34.7	
24	3595	32.6	
**Residence**			0.000***
Urban	6680	35.3	
Rural	14,139	41.1	
**Education level**			0.000***
None	7207	49.6	
Primary	7993	43.5	
Secondary	5432	28.3	
Tertiary	183	14.6	
**Wealth status**			0.000***
Poor	9731	43.7	
Middle	4303	39.5	
Rich	6785	33.6	
**Employment status**			0.005**
Not working	7593	38.4	
Working	12,376	40.1	
**Knowledge of any FP method**			0.000***
Knows no method/traditional method	1321	56.1	
Knows modern method	19,498	38.2	
**Age at first marriage**			0.000***
Less than 15 years	6160	89.6	
15–19 years	11,008	37.1	
20–24 years	1045	13.2	
**Age at first sex**			0.000***
Less than 15 years	6650	75.5	
15–24 years	11,030	33.7	
**Household size**			0.000***
1–3	3563	27.2	
4–5	8215	46.3	
**6+**	9040	40.2	
**Ideal number of children**			0.000***
0	394	42.6	
1–3	3908	27.7	
4–5	8728	39.1	
6+	6332	49.5	
**Visited by community health worker**			0.688^ns^
No	15,917	38.7	
Yes	2848	39.0	
**Exposure to media FP messages**			0.000***
No	13,718	41.3	
Yes	7101	35.3	
**Total**	**20,819**	39.0	

*** =*p* < 0.001; ns = not significant

### Factors associated with early childbearing among young women

Table 3 presents multivariable logistic regression results. A young woman’s level of education; place of residence, age at first sex; desired family size and household size were significantly associated with early childbearing in the pooled analysis for 31 SSA countries. Conversely, household wealth status, knowledge of family planning, employment status, being visited by a community health worker, and exposure to mass-media family planning messages were not associated with adolescent childbearing. Increased level of education was associated with reduced odds of early childbearing among young women. Young women with secondary (aOR = 0.57; 95% CI: 0.52, 0.62) or tertiary level of education (aOR = 0.32; 95% CI: 0.22, 0.45) were less likely to experience early childbearing.

Increasing age at first sexual debut reduced the chance of early childbearing. Results show that young women who first had sexual intercourse in the age range of 15–24 years had lower odds of experiencing early childbearing (aOR = 0.15; 95% CI: 0.14, 0.16). Furthermore, young women who desired 1–3 children (aOR = 0.63; 95% CI: 0.58, 0.69) had lower odds of experiencing early childbearing. On the other hand, belonging to a household with more than 3 members was associated with increased chance of early childbearing among young women. Those who belonged to households with 4–5 members (aOR = 2.13; 95% CI: 1.97, 2.31) or 6 or more members (aOR = 1.68; 95% CI: 1.55, 1.81) had higher odds of experiencing early childbearing compared to young women from households with fewer members.

**Table 3:** Adjusted odds ratios for the binary logistic regression of the association between background characteristics and early childbearing, SSA DHS Data (N = 54,671)

Early childbearing			
Background Characteristics	aOR	p-value	95% CI
Age			
20	**1**		
21	0.83	0.000***	0.75–0.92
22	0.75	0.000***	0.69–0.82
23	0.63	0.000***	0.58–0.69
24	0.53	0.000***	0.48–0.58
**Residence**			
Urban	**1**		
Rural	0.91	0.021*	0.83–0.98
**Education level**			
None	**1**		
Primary	0.93	0.059^ns^	0.86–1.00
Secondary	0.57	0.000***	0.52–0.62
Higher	0.32	0.000***	0.22–0.45
**Wealth status**			
Poor	**1**		
Middle	0.93	0.056^ns^	0.86–1.00
Rich	0.94	0.166^ns^	0.86–1.03
**Knows any FP method**			
Knows no method/traditional method	**1**		
Knows modern method	1.05	0.623^ns^	0.87–1.26
**Employment status**			
Not working	**1**		
Working	1.03	0.342^ns^	0.97–1.10
**Age at first sex**			
Less than 15 years	**1**		
15–24 years	0.15	0.000***	0.14–1.16
**Household size**			
1–3	**1**		
4–5	2.13	0.000***	1.97–2.31
**6+**	1.68	0.000***	1.55–1.92
**Desired family size**			
**1–3**	0.63	0.000***	0.58–0.69
**4–5**	0.85	0.000***	0.78–0.92
**6+**	**1**		
**Visited by community health worker**			
No	**1**		
Yes	0.97	0.534^ns^	0.89–1.06
**Exposure to media FP messages**			
No	**1**		
Yes	0.99	0.778^ns^	0.93–1.05

*** *p* < 0.001; * = *p* < 0.05; ns = not signifcant

## Discussion

Early childbearing has been a significant barrier to reducing fertility in SSA [4, 6, 14, 23]. This is because early childbearing often leads to adolescent girls dropping out of school or being unable to pursue higher education. The lack of education can limit adolescents’ access to information and services for family planning, making it harder for them to make informed decisions about their reproductive health such as limiting or spacing children.

This study was conducted using pooled DHS data for 31 countries in sub-Saharan Africa to better understand the factors associated with early childbearing. Study results revealed that the prevalence of women who experienced early childbirth in SSA was still high at 39.0% (95% CI: 35, 43). This finding is similar to what has been reported by previous studies. A study of 2021 by Melesse and others reported a prevalence of 47% in SSA [47]. UNICEF in 2021 estimated that 26.7% of women aged 20–24 experienced early child bearing in SSA [52]. Literature also shows that there are country variations in the prevalence of early childbearing in SSA. Avogo and Somefun in 2019 found that 13% of Nigerian adolescents, 12% in Burkina Faso, and 27% in Niger have had a first birth [46]. Wado and others in 2019 found that the prevalence of early motherhood ranged from 18% in Kenya to 29% in Malawi and Zambia [3].

Similar to what has been reported in literature, this study shows that Rwanda had the lowest proportion of adolescent births 8.4% (95% CI: 7, 9) while Chad had the highest prevalence at 58% (95% CI: 56, 59). The high prevalence of early childbirth observed in Chad confirms the findings of a similar study conducted by Ahinkora (2021) in 32 SSA countries, which also found that Chad had the highest prevalence of adolescent pregnancy (76.6%) while Rwanda had the lowest at 9.2% [53]. The low prevalence of early childbirth in Rwanda could be attributed to the high prevalence of contraceptive use among adolescents, while the high prevalence in Chad could be explained by low contraceptive use among adolescents [54, 55]. Furthermore, Sara (2020) found that Chad, Niger, and Benin had the highest proportions of adolescents who gave birth before the age 16 [6]. Our study further found that a young woman’s education level, age at first sex, family size desire and household size were significantly associated with early childbearing in SSA countries.

Several studies have reported the association of education with early childbearing and other reproductive health outcomes [9, 28, 32, 49, 50, 53, 56, 57] in SSA and elsewhere. Literature shows that increasing a woman’s education is one factor that has been associated with improved contraception uptake, hence reducing the risk of teenage pregnancy [28, 32, 58, 59]. In this current analysis, women with a secondary or tertiary level of education were less likely to experience early childbirth. This suggests that increased schooling opportunities for women have the potential to further reduce the high prevalence of early childbirth in SSA. This is because educated women have the potential to make an informed decision about delaying the onset of sexual relationships, averting early marriages and can influence contraception uptake beacause of easy access to appropriate reproductive health information as well understanding of fundamental child rights [60]. Our finding is consistent with earlier studies in SSA, Asia, and other parts of the world [53, 57, 61–66] which also reported education as a significant factor in reducing exposure to early childbirth, child marriage as well as early sexual debut. Additionally, literature shows that women whose partners had secondary or higher education are less likely to experience early childbirth compared to those whose partners have a lower level of education [56, 67].

Our findings reveal that a higher age at first sexual debut was significantly associated with lower rates of early childbirth in SSA. This finding is supported by earlier studies conducted elsewhere in the world [68–70]. The fact that delaying sexual debut is linked to lower exposure to teenage pregnancy could be the possible explanation for this finding [71]. Other possible explanations include the fact that older adolescent girls may be better able to negotiate safer sex with their partners and hence increase the chances of frequent and effective use of contraceptives to avoid pregnancy [53, 69, 72].

Lastly, findings show that adolescents from the households with 4 members or more were highly likely to experience early childbirth compared to their counterparts from households with 3 members or fewer. It is anticipated that households with 4 members or more may not have adequate resources to support access to education, especially for a girl child; hence, girls from these families are less likely to access and comprehend health education messages leading to a low understanding of the consequences of early childbirth and marriage [73–75]. Therefore, strategies for preventing teenage childbirth should not only be directed to teens themselves but also consider household-level contextual factors that fall into two general categories: empowering parents with sexual and reproductive health information; and encouraging open discussions about SRH issues between parents and girl children [4, 53, 75, 76].

Increased access to education for female adolescents and young women together with strengthening access to sexual reproductive health information through social media and community-based interventions will be key to addressing the problem of adolescent child bearing in SSA. As evidenced by the results, women with a secondary or higher levels of education were less likely to have experienced early childbirth, suggesting that keeping girls in school is a significant factor in reducing adolescent fertility. Early sexual debut is another deterrent factor to reducing adolescent fertility in SSA. There is a need for deliberate policy actions aimed at integrating comprehensive sexual education into the early school curriculum and a thorough community profiling to identify cultural barriers that impede girls from advancing their education prospects. Furthermore, interventions to curb adolescent childbearing require approaches that will strengthen SRH programming through community engagement among relevant stakeholders such as parents, teachers, civic leaders, traditional leaders, community leaders, and religious institutions.

This study utilised the theoretical underpinnings of the Classical Demographic Transition Theory and the Empowerment Theory. The two theories have provided the framework for understanding how individual and household socio-economic factors have influenced early childbearing experienced among women in SSA. In this regard, the study shows that having formal education reduces the risk of early childbearing among women. Therefore, the findings of the study affirm that empowering women through education opportunities has the potential to reduce the prevalence of early childbearing in SSA. Education attainment for women in this sense can be viewed as an empowerment tool to help young women get employment opportunities which in turn can enhance access to sexual reproductive health services. Access to sexual reproductive health services will enable women to make informed choice about meeting their reproductive goals. The findings of this current study have significant implications for the theoretical understanding of determinants of high fertility in SSA. Furthermore, the findings are significant for strengthening of sexual reproductive health interventions to prevent adolescent pregnancies in the region. Thus, the evidence generated by this study will guide health policymakers in designing health policies and interventions that address the unique sexual reproductive health care needs of adolescent girls in the region.

Although the study has provided useful findings that have the potential to inform the strengthening of existing sexual reproductive policies and programming targeting at changing reproductive health behaviour among women in SSA, designing of tailor-made SRH interventions to address country-level specific fertility problems will require a detailed decomposition analysis of both individual and community factors to delineate factors that explain heterogeneity in the observed prevalence of early childbirth across countries in SSA. Countries that have a high prevalence of adolescent fertility may consider adopting and customising SRH policies for countries where the problem is minimal.

### Study strengths and limitations

Since the study comprised nationally representative samples of women from 31 countries in SSA, the current findings can apply to the entire population of women in the age range 20–24 years in the region. Our study has contributed to the literature by conducting a comprehensive examination of pooled data. This has enhanced a holistic understanding of the factors that affect the reproductive decision behaviour of young women using recent demographic data. However, it is important to note that a good number of countries in SSA do not have recent DHS data, thus making our findings not able to present a comprehensive recent picture of early childbearing in SSA. Additionally, because of the cross-sectional nature of the DHS data, causality cannot be inferred from this study. There is also a possibility of recall bias, since the DHS participants were asked to report events that happened in the past. The study could not bring out factors that explain observed differentials in the prevalence of early childbearing because of divergent socio-cultural factors prevailing in the region.

## Conclusion

Even though fertility is slowly declining in SSA, the prevalence of early childbearing is still high in the region. This study has established significant variations in the prevalence of early childbearing across SSA countries and sub-regions. Age at first sexual debut, level of education, household size, and desired family size are associated with early childbearing in SSA. This calls for country-specific targeted sexual reproductive health policies and interventions to address early childbearing. Furthermore, our results provide evidence for strengthening the provision of comprehensive sexual reproductive health information and services through primary school curriculum and community interventions in countries where the prevalence of early childbearing is high. Further research is needed to expand this current analysis by investigating the linkages between beliefs, social norms and fertility desire with early childbearing among young women in SSA.

## Data Availability

Data used in our study is publicly available at DHS program website (https://dhsprogram.com/).

## Abbreviations

CI: Confidence Interval
DHS: Demographic and Health Survey
EA: Enumeration Area
FP: Family Planning
SDG: Sustainable Development Goal
SRH: Sexual Reproductive Health
SSA: Sub-Saharan Africa
UNFPA: United Nations Population Fund
UNICEF: United Nations Children Fund
USAID: United States Aid for International Development
WHO: World Health Organisation
